# Adverse Childhood Experiences and Risk of Abnormal Body Mass Index: A Global Systematic Review and Meta-Analysis

**DOI:** 10.3390/children11081015

**Published:** 2024-08-20

**Authors:** Sohrab Amiri, Nailah Mahmood, Rahemeen Yusuf, Nadirah Ghenimi, Syed Fahad Javaid, Moien AB Khan

**Affiliations:** 1 Medicine, Quran and Hadith Research Center, Baqiyatallah University of Medical Sciences, Tehran 17166, Iran; rsr.amiri.s@bmsu.ac.ir; 2 Division of Health Research, Lancaster University, Lancaster LA1 4YW, UK; n.mahmood2@lancaster.ac.uk; 3 Emirates Center for Happiness Research, United Arab Emirates University, Al-Ain 15551, United Arab Emirates; r.yusuf@uaeu.ac.ae; 4 Health and Wellness Research Group, Department of Family Medicine, College of Medicine and Health Sciences, United Arab Emirates University, Al-Ain 15551, United Arab Emirates; nghenimi@uaeu.ac.ae; 5 Health and Wellness Research Group, Department of Psychiatry and Behavioral Sciences, College of Medicine and Health Sciences, United Arab Emirates University, Al-Ain 15551, United Arab Emirates

**Keywords:** adverse childhood experiences, body mass index, obesity, overweight, systematic review, meta-analysis

## Abstract

(1) Objectives: The impact of abnormal body mass index (BMI) on health is extensive, and various risk factors contribute to its effects. This study aimed to examine the association between adverse childhood experiences (ACEs) and BMI categories, including underweight, overweight, obesity, severe obesity, and morbid obesity; (2) Methods: Three databases were searched: Web of Science, PubMed, and Scopus. Manual searches were conducted using Google Scholar and ResearchGate. Odds ratios (ORs) and 95% confidence intervals (CIs) were calculated to assess the association between ACEs and BMI. A random-effects model was used to combine the ORs and CIs across studies; (3) Results: This meta-analysis included 71 studies. The pooled ORs for the relationship between ACEs and obesity was 1.42 (95% CI: 1.24–1.63, Z = 4.96, *p* < 0.001), indicating a significant association. ACEs showed a positive association with overweight (OR = 1.16, 95% CI: 1.06–1.27, Z = 3.24, *p* = 0.001). Specifically, ACEs ≥ 4 were strongly associated with obesity (OR = 2.06, 95% CI: 1.27–3.36, Z = 2.90, *p* = 0.004). Sexual abuse was also found to be significantly associated with obesity (OR = 1.46, 95% CI: 1.29–1.65, Z = 5.98, *p* < 0.001); (4) Conclusion: This study finds that individuals who have experienced ACEs are more likely to have a higher BMI in adulthood. Therefore, ACEs should be considered a factor associated with abnormal BMI.

## 1. Introduction

The term ‘adverse childhood experiences’ (ACEs) covers a wide range of significant stressors that can profoundly impact the development of a child. They are defined as potentially traumatic and disrupted child experiences affecting childhood between the age range of (0–17 years) [1]. Over the years ACE has become a significant public concern due to its effect on the children’s physical, emotional, and cognitive well-being, which is well-documented in scientific research [2,3]. It has been estimated that between 1.1% and 6% of a nation’s gross domestic product is spent on addressing the negative consequences of ACEs [4,5]. Adversities such as abuse, neglect, household dysfunction, and other adversities are considered stressors during childhood [2]. Several studies have shown that ACEs can have long-term impacts on the physical and mental health of the child [6,7,8,9,10,11]. Children who are raised in dysfunctional families or poverty-stricken areas are more vulnerable to experiencing ACE [12]. One of the main risk factors of ACE is poor child and caregiver attachment bond, which can act as a buffer to the negative effect of ACE. Children with poor caregiver–child relationships are more vulnerable to eating disorders, a major health concern. A strong, stable, and healthy caregiver–child relationship is crucial for building resilience, which helps prevent eating disorders by enabling individuals to bounce back from adverse life situations [13,14]. Resilience can help mitigate the negative effects of ACE due to which individuals achieve positive outcomes, despite these traumatic experiences [15].

ACEs significantly affect health later in life [4,5]. The effects of ACEs on health—such as chronic diseases, cancer development, mental health conditions, self-rated health, HIV risk behaviour, and eating disorders—have been studied in various countries [16,17,18,19,20,21]. Underweight, overweight, and obesity are associated with negative consequences throughout a person’s life, as is an abnormal body mass index (BMI) [22,23]. Numerous studies have indicated that the risk of obesity in adults is rooted in adversity in early child development [24,25,26]. Globally, the prevalence of obesity has been steadily increasing, with the World Health Organization [27] reporting more than 650 million obese individuals (BMI 30 kg/m^2^). The studies reported that 39% of adults aged 18 years and older were overweight and 13% were obese [27]. In addition, 9% of the population were underweight [23,28].

The association between an abnormal BMI and adverse health outcomes has been well established. Millions of deaths have been attributed to high BMI, resulting from an increased risk of morbidity and mortality [29]. Moreover, abnormal BMI is associated with a range of diseases, including hypertension, chronic obstructive pulmonary disease, coronary heart disease, cancer, disability retirement, stroke, Alzheimer’s disease, and mental disorders [30,31,32,33,34,35].

Many studies have examined the risk factors associated with underweight, overweight, and obesity because of their significant impact on health outcomes. There is also a modest association between ACEs and overweight [36]. Studies have shown that ACEs are associated with an increased risk of underweight [37,38], which implies that ACEs can be related not only to underweight but also to unhealthy weight control behaviours [38]. Several reviews have suggested that ACEs contribute to an abnormal BMI [25,36,39,40,41,42,43,44,45,46]. The odds ratios (ORs) of previous meta-analyses investigating the association between ACEs and BMI ranged from 1.34 to 1.36 [39,40,46].

ACEs and BMI have been the subjects of increasing research interest. However, several important issues require further investigation. ACE and BMI studies have primarily focused on specific ACE components [41,42], potentially overlooking the comprehensive impact of ACEs. The scope of previous meta-analyses was limited, often including fewer original studies; for example, 18 [36], 23 [40], and 24 [44] studies. The relationship between ACEs and underweight, severe obesity, and morbid obesity has not been thoroughly explored [25,36,39,40,41,42,43,44,45,46], with most research focusing on the relationship between ACEs and obesity. However, studies in this field are expanding, and new scientific evidence has been found; therefore, it is necessary to update this field. This study aims to address these gaps in the literature by examining three main relationships: the relationship between ACEs and BMI classes, the relationship between different ACEs and BMI classes, and the relationship between the number of ACEs and BMI classes.

## 2. Methods

### 2.1. Search Strategy

**Figure 1:**
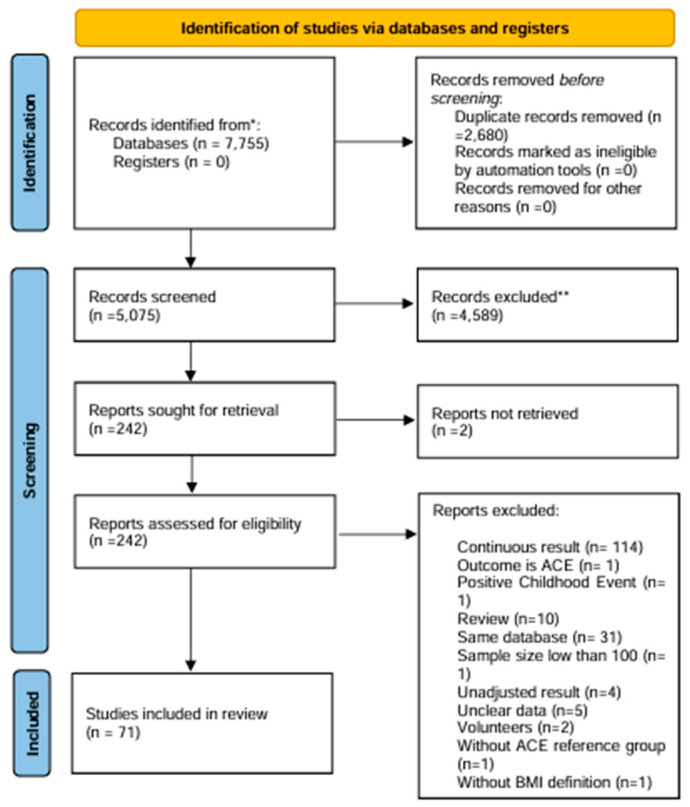
Flowchart diagram.

This systematic review followed the Preferred Reporting Items for Systematic Reviews and Meta-analyses [47] and the Cochrane Handbook for Systematic Reviews of Interventions [48]. The protocol for this systematic review was registered with The International Register of Systematic Reviews (PROSPERO) under the registration number CRD42023396788. This review did not require ethics committee approval or informed consent from the study population because the data were obtained from open-source databases and Internet searches. A flow diagram of the study selection process is shown in Figure 1.

We developed a search strategy using a predefined set of keywords and searched Web of Science, PubMed, and Scopus using this strategy. A manual search of Google Scholar and ResearchGate was also conducted as these databases include grey literature in addition to peer-reviewed journal articles. The search was conducted in English from the inception of the databases until August 2023. Appendix A presents the syntax of the keywords used. In addition, the full texts of previous reviews were collected, and references were reviewed.

### 2.2. Inclusion and Exclusion Criteria

To be included in this review, studies had to meet the following criteria: (1) report the association between ACEs and BMI, (2) have a cross-sectional, retrospective, prospective cohort, or case-control study design, (3) include a target sample size of at least 100 participants to minimise selection bias, (4) have a reference group without ACEs, (5) report results with a 95% confidence interval, odds ratio, risk ratio, or relative risk, and (6) provide adjusted results on the relationship between the exposure variable (ACEs) and outcome (BMI). Studies reporting continuous results were excluded because they did not allow for a clear interpretation of effect size. Studies with voluntary or referral populations and those lacking sufficient results for adjusted odds ratio were also excluded. In cases in which multiple reports were published from the same database, only one study with the highest quality (including the highest sample size, regression analysis, subgroup analysis, and highly adjusted results) was selected.

### 2.3. Outcome and Exposure Definition

The outcome variable in this systematic review was BMI. Based on the existing standards in BMI classification, the following classes were included in this study: underweight (BMI ≤ 18.5 kg/m^2^), overweight (BMI 25–29.9 kg/m^2^), obesity (BMI 30–34.9 kg/m^2^), severe obesity (BMI 35–39.9 kg/m^2^), and morbid obesity (BMI ≥ 40 kg/m^2^).

ACEs include a series of events, as discussed by Felitti et al. [4], such as sexual, physical, and emotional abuse and neglect.

### 2.4. Data Extraction

To extract data, authors, publication year, country, study design, sex and age of participants, sample size, measurement methods for exposure and outcome variables, ORs or risk ratios with 95% confidence intervals (CIs), and adjusted variables were collected for each eligible study. Supplementary Table S1 provides detailed information.

### 2.5. Quality Assessment

The quality assessment of the included studies was conducted using the Effective Public Health Practice Project Quality Assessment Tool. This tool evaluates studies based on five dimensions [49,50]. Definitions for each dimension are provided in Appendix B.

### 2.6. Statistical Analysis

The effect sizes (odds ratio, risk ratio, or relative risk) between ACEs and BMI were extracted from all included studies. Using the fixed effects method, studies with multiple independent effect sizes were converted to pooled effect sizes. Using random effects, ORs and 95% CIs of the studies were combined. An analysis was conducted to examine the relationship between ACEs and underweight, overweight, obesity, severe obesity, and morbid obesity. The number of ACEs (cumulative ACEs) and types of abnormal BMI were analysed. The types of ACEs and their relationship with abnormal BMI were also examined.

Using the pooled effect size and the standard error of this effect size, we evaluated heterogeneity using chi-squared and I^2^ [51,52] and publication bias using a funnel plot, Egger’s test, and the trim-and-fill method [53,54,55]. Stata 14 software (Stata Corp., College Station, TX, USA) and comprehensive meta-analysis version-3 [56] were used for the data analysis.

### 2.7. Selection Process

The studies were screened based on the flowchart shown in Supplementary Figure S1. The screening included three steps: identification, screening, and inclusion. Based on the screening steps detailed in Supplementary Figure S1, 71 studies [16,26,37,38,57,58,59,60,61,62,63,64,65,66,67,68,69,70,71,72,73,74,75,76,77,78,79,80,81,82,83,84,85,86,87,88,89,90,91,92,93,94,95,96,97,98,99,100,101,102,103,104,105,106,107,108,109,110,111,112,113,114,115,116,117,118,119,120,121,122,123] were finally included in the meta-analysis.

### 2.8. Quality of Studies

The results of the qualitative assessment of the studies included in this meta-analysis are listed in Supplementary Table S1, and the five dimensions of quality assessment are shown.

## 3. Results

### 3.1. Systematic Review

Our thematic analysis [50], involved three stages: initial coding, grouping codes into descriptive themes, and refining them into analytical themes. This structured methodology allowed for a comprehensive synthesis of complex data on the impact of ACEs on BMI [124].

### 3.2. Prevalence and Severity of ACEs Linked to Abnormal BMI

The prevalence and severity of ACEs have been strongly linked to an increased risk of abnormal BMI in various studies. Studies have demonstrated that experiences of childhood sexual abuse (CSA), physical abuse, neglect, and other forms of maltreatment are significantly correlated with higher BMI in adulthood, indicating a dose–response relationship where the severity and intensity of maltreatment are directly proportional to the risk of obesity [57,59,69,72]. This relationship persists even after adjusting for demographic factors and lifestyle behaviours, emphasising the profound effects of childhood maltreatment on weight-related health outcomes. Conversely, the relationship between ACEs and underweight has been less explored, suggesting the need for further research to fully understand how specific ACEs may affect BMI differently [74,75]. These findings highlight the critical need for targeted interventions to address both ACEs and their long-term health impacts, underscoring the strong association between various forms of ACEs and an increased risk of abnormal BMI.

### 3.3. Sex and Socioeconomic Factors Modulating the ACE–BMI Relationship

Sex and socioeconomic factors significantly influence the relationship between ACEs and abnormal BMI. The review indicates sex-specific outcomes in which women are more susceptible to severe obesity linked to emotional neglect, whereas men may exhibit different or less pronounced associations between ACEs and BMI [75,78,101,117,123]. Furthermore, experiences of physical and emotional abuse are particularly associated with an increased risk of obesity, with specific populations, such as gay men who have experienced CSA, facing compounded risks [59,69,72,83,84,85,106,107,108]. While all types of ACEs contribute to the risk of developing abnormal BMI, CSA, physical abuse, and neglect have been repeatedly identified for their specific impact on the likelihood of obesity. These sex differences suggest that the psychological impact of trauma may vary, affecting body image and health behaviours differently across sexes. Furthermore, socioeconomic variables, such as family income and parental education along with environmental factors such as parental unemployment and witnessing domestic violence, have been identified as crucial in modulating the effects of ACEs on BMI. These factors can either exacerbate or mitigate obesity risks and engagement in extreme weight loss behaviours, indicating the nuanced ways in which ACEs affect health outcomes beyond direct psychological effects [37,38,73,87,116,120].

### 3.4. Psychological Mediators and Comorbid Health Behaviours

Psychological mediators and comorbid health behaviours significantly influence the relationship between ACEs and abnormal BMI. This review identifies that mental health issues and health-risk behaviours—such as smoking, alcohol use problems, and insufficient physical activity—play a critical role in mediating the association between childhood abuse and adult obesity. Addressing mental health and modifying health-risk behaviours are essential components of interventions designed to mitigate the long-term impact of ACEs on BMI [62,68,94,95,111,112]. Further studies highlight the importance of psychological distress and unhealthy behaviours as significant mediators in the ACE–BMI relationship, highlighting how emotional neglect and abuse contribute to obesity and cardiometabolic risk factors.

### 3.5. The Cumulative Effect of ACEs on Health Outcomes

Studies have demonstrated that individuals with higher ACE scores are notably more likely to face health and behavioural outcomes associated with abnormal BMI, such as diabetes, hypertension, and obesity, underscoring the dose-response relationship between the severity and number of ACEs and the increased risk of overweight and obesity. This relationship suggests that the cumulative impact of multiple ACEs markedly increases health risks, emphasising the need for comprehensive interventions that address the broad spectrum of childhood adversities to effectively mitigate these risks [16,64,86,95,97,99,100,113,115]. Furthermore, the literature indicates the substantial societal burden of ACEs, including their significant contribution to high BMI and other health risks, with specific types of ACEs showing strong associations with obesity.

### 3.6. Meta-Analysis

#### 3.6.1. ACEs and Obesity

**Figure 2:**
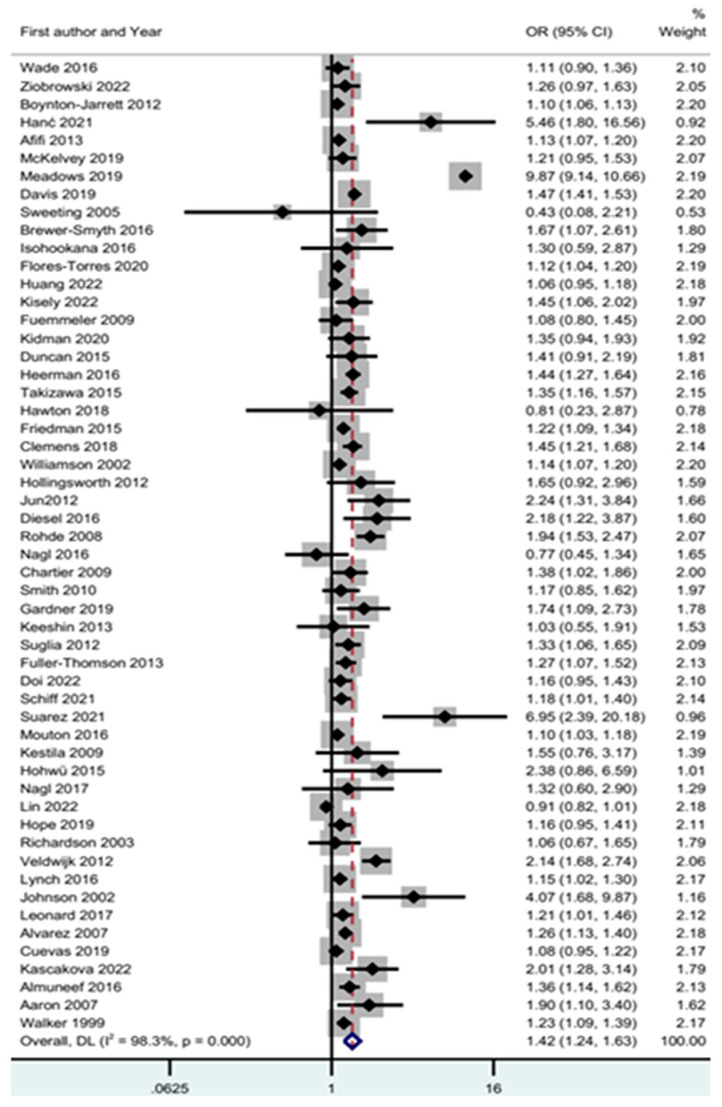
Meta-analysis of adverse childhood experiences and obesity.

Figure 2 displays a meta-analysis of adverse childhood experiences and obesity, showing the relationship between ACEs and obesity. The relationship between ACEs and obesity was (OR = 1.42; CI 1.24–1.63; Z = 4.96; *p* < 0.001; I^2^ = 98.3%).

#### 3.6.2. ACEs and Overweight and Overweight/Obesity

**Figure 3:**
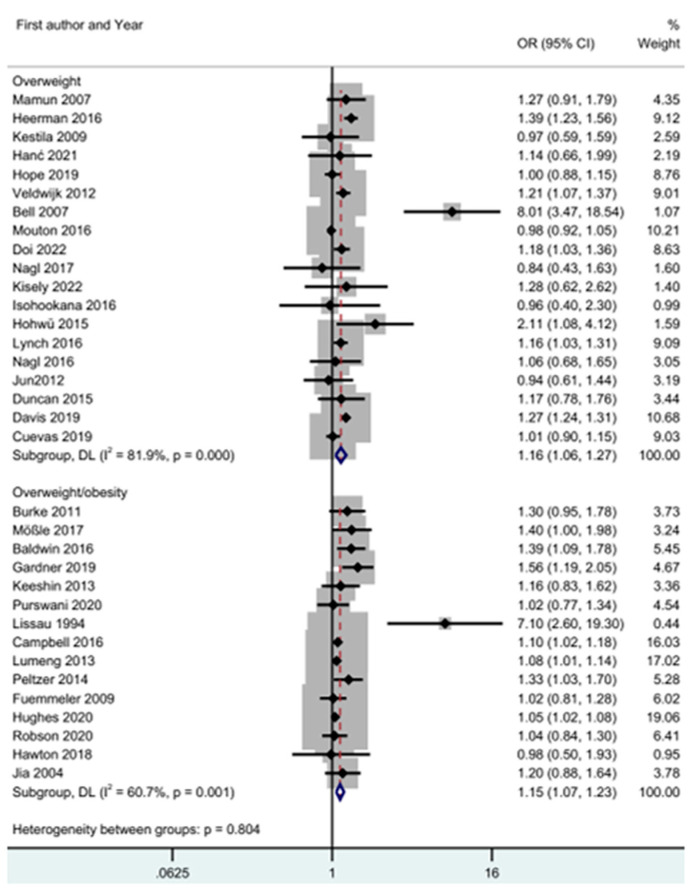
Meta-analysis of adverse childhood experiences and overweight and overweight/obesity.

Figure 3 displays a meta-analysis of adverse childhood experiences and overweight and overweight/obesity, showing the relationship between ACEs and overweight and overweight/obesity. The relationship between ACEs and overweight was (OR = 1.16; CI 1.06–1.27; Z = 3.24; *p* = 0.001; I^2^ = 81.9%). The relationship between ACEs and overweight/obesity was (OR = 1.15; CI 1.07–1.23; Z = 3.99; *p* < 0.001; I^2^ = 60.7%).

#### 3.6.3. ACEs and Morbid Obesity, Severe Obesity, and Underweight

Supplementary Figure S2 shows the relationship between ACEs and morbid obesity, severe obesity, and underweight. The relationship between ACEs and morbid obesity was (OR = 1.26; CI 1.13–1.41; Z = 4.08; *p* < 0.001; I^2^ = 16.1%). The relationship between ACEs and severe obesity was (OR = 1.47; CI 1.20–1.80; Z = 3.76; *p* < 0.001; I^2^ = 94.5%). The relationship between ACEs and underweight was positive and insignificant.

#### 3.6.4. ACEs 1 and Underweight, Overweight, Obesity, Severe Obesity, and Morbid Obesity

Supplementary Figure S3 shows the relationship between the number of ACEs equal to 1 (only one ACE) for various classes of BMI. These relations were non-significant for underweight, overweight/obesity, and obesity; for overweight, the relationship was (OR = 1.15; CI 1.05–1.26; Z = 3.02; *p* = 0.003; I^2^ =5 8.6%); for severe obesity, the relationship was (OR = 1.09; CI 1.06–1.58; Z = 2.53; *p* = 0.011; I^2^ = 86.6%); for morbid obesity, the relationship was (OR = 1.24; CI 1.08–1.42; Z = 2.98; *p* = 0.003; I^2^ = 0%).

#### 3.6.5. ACEs 2 and Underweight, Overweight, Obesity, and Severe Obesity

Supplementary Figure S4 shows the relationship between the number of ACEs equal to 2 and various BMI classes. This relation was only significant for severe obesity (OR = 1.41; CI 1.11–1.78; Z = 2.85; *p* = 0.004; I^2^ = 86.3%).

#### 3.6.6. ACEs 3 and Underweight, Overweight, Obesity, and Severe Obesity

Supplementary Figure S5 shows the relationship between the number of ACEs equal to 3 and various BMI classes. This relation was only significant for severe obesity (OR = 1.56; CI 1.31–1.86; Z = 5.01; *p* < 0.001; I^2^ = 63.9%).

#### 3.6.7. ACEs ≥ 4 and Underweight, Overweight, Obesity, Severe Obesity, and Morbid Obesity

Supplementary Figure S6 shows the relationship between number of ACEs ≥ 4 with various BMI classes. These relations were only significant for obesity (OR = 2.06; CI 1.27–3.36; Z = 2.90; *p* = 0.004; I^2^ = 98.2%) and for severe obesity (OR = 1.77; CI 1.31–2.38; Z = 3.72; *p* < 0.001; I^2^ = 91.3%).

#### 3.6.8. Emotional Abuse and Emotional Neglect and BMI Class

Supplementary Figure S7 shows the relationship between emotional abuse, emotional neglect, and BMI class. The relationship between emotional abuse with overweight was positive and not significant; for obesity, it was (OR = 1.67; CI 1.23–2.26; Z = 3.29; *p* = 0.001; I^2^ = 78.3%). The relationship between emotional neglect with underweight and overweight was positive and insignificant; for obesity, it was (OR = 1.38; CI 1.23–1.56; Z = 5.28; *p* < 0.001; I^2^ = 0%).

#### 3.6.9. Physical Abuse and BMI Class

Supplementary Figure S8 shows the relationship between physical abuse and BMI class. The relationship between physical abuse with overweight and overweight/obesity was positive and insignificant; for obesity, it was (OR = 1.28; CI 1.16–1.40; Z = 5.10; *p* < 0.001; I^2^ = 80.1%). The relationship between physical abuse with underweight was (OR = 1.20; CI 1.05–1.38; Z = 2.60; *p* = 0.009; I^2^ = 0%).

#### 3.6.10. Physical Neglect and BMI Class

Supplementary Figure S9 shows the relationship between physical neglect and BMI class. The relationship between physical neglect with overweight and underweight was insignificant; for obesity, it was (OR = 1.58; CI 1.09–2.28; Z = 2.41; *p* = 0.016; I^2^ = 55.6%).

#### 3.6.11. Sexual Abuse and BMI Class

Supplementary Figure S10 shows the relationship between sexual abuse and BMI class. The relationship between sexual abuse with overweight, morbid obesity, and underweight were insignificant; for obesity, it was (OR = 1.46; CI 1.29–1.65; Z = 5.98; *p* < 0.001; I^2^ = 83%), for overweight/obesity, it was (OR = 1.25; CI 1.04–1.49; Z = 2.44; *p* = 0.014; I^2^ = 48.4%). The relationships between ACEs and other BMI classes were insignificant.

#### 3.6.12. Publication Bias and Heterogeneity

Publication bias is shown in Supplementary Figure S11. Evaluating the association between ACEs and obesity revealed a high heterogeneity, with an I^2^ value of 98.3% [51,125]. The heterogeneity chi-square test was 3050.25 (d.f = 53; *p* < 0.001)31. The Egger (*p* = 0.572) test did not show any publication bias. The trim-and-fill [55] was intended to identify missing studies in the meta-analysis and provide adjusted results. Its results showed that there were 11 missing studies, and the odds ratio was equal to 1.55, with a CI 1.37–1.76. 

Publication bias is shown in Supplementary Figure S12. For the evaluation of heterogeneity in the association between ACEs and overweight status, the I^2^ was 81.9%. This indicator showed a high heterogeneity [51]. The heterogeneity chi-square test was 99.23 (d.f = 18; *p* < 0.001). The Egger (*p* = 0.534) test showed publication bias [55]. The trim-and-fill test was intended to identify missing studies in the meta-analysis and to provide adjusted results. Its results showed that there were two missing studies, and the odds ratio was equal to 1.18, with a CI of 1.08–1.29.

Publication bias is shown in Supplementary Figure S13. For the evaluation of heterogeneity in the association between ACEs and overweight/obesity, I^2^ was 60.7%. This indicator depicted a moderate heterogeneity [51]. The heterogeneity chi-square test was 35.66 (d.f = 14; *p* = 0.001)31. The Egger (*p* = 0.005) test showed publication bias. The trim-and-fill [55] test was intended to identify missing studies in the meta-analysis and provide adjusted results. Its results showed that there were five missing studies, and the odds ratio was equal to 1.07, with a CI of 1.00–1.16.

## 4. Discussion

This systematic review and meta-analysis highlighted a significant association between ACEs and an increased risk of abnormal BMI, including underweight, overweight, obesity, severe obesity, and morbid obesity. Our findings corroborate with previous research indicating a dose–response relationship between the prevalence and severity of ACEs—such as CSA, physical abuse, neglect, and other forms of maltreatment—and higher BMI in adulthood [57,59,69,72]. This relationship persists across demographic factors and lifestyles, emphasising the profound impact of childhood maltreatment on weight-related health outcomes.

The intricate association between ACEs and BMI abnormalities involves a myriad of interconnected pathways. Central to this relationship is the concept of a thrifty phenotype, precipitated by ACEs, which instigates metabolic alterations in children, inclining them towards reduced energy expenditure and heightened susceptibility to overconsumption of food [126,127]. Hormonal imbalances, particularly cortisol dysregulation stemming from stressors, contribute significantly to adipose tissue deposition, thereby augmenting the risk of overweight status [128]. Furthermore, epigenetic modifications in the genes governing glucose and fat metabolism exacerbate these metabolic perturbations, intensifying the propensity for abnormal BMI trajectories [129].

The neurobiological underpinnings of emotion regulation also play a prominent role in elucidating the ACE–BMI nexus [130]. Early traumatic experiences disrupt the development of the neurobiological architecture, impairing emotion regulation and resilience to subsequent stressors. This, in turn, fosters maladaptive coping mechanisms such as emotional eating, exacerbating the risk of obesity [37]. Moreover, ACEs may precipitate the development of eating disorders and addictions, further entrenching alterations in body weight regulation [131].

Furthermore, insights from other studies [21] underscore the pivotal role of ACE-influenced lifestyle choices in preventing obesity and weight gain.

A poor parental attachment and bond is also one of the consequences of adverse childhood experiences. When parents do not develop a bond with children where they can positively communicate and discuss sensitive issues such as disordered eating habits, the chances of an increase in unhealthy eating behaviours are higher [132]. Unlike in a parental–child relationship where parents can monitor their children’s eating habits and provide them with effective nutritional advice [133]. Also, adverse childhood experiences undermine resilience, the ability through which individuals can bounce back from complex situations [15]. The higher the resilience the more likely it is for the individual to recover from eating disorders. The reason being resilient individuals were seen to interact more with other resilient individuals or those who have positive mental states that helped have a positive influence on their eating behaviour [134].

ACEs induce pervasive stress responses in children, which have enduring implications on adult weight status [135,136]. Biological aberrations arising from ACEs disrupt energy homeostasis and nutritional behaviours, perpetuating the cycle of weight gain. The relatively nascent exploration of the association between ACEs and underweight status accentuates the nuanced nature of their impact on BMI. This underscores the imperative for tailored interventions that account for sex differentials, socioeconomic gradients, and the integration of mental health and lifestyle modifications into holistic care paradigms [126,137]. The relationship between ACEs and BMI abnormalities is multi-faceted and governed by intricate biological, psychological, and social determinants. Unravelling these complex pathways is imperative for informing targeted interventions aimed at ameliorating the enduring health sequelae of childhood adversity.

A dose–response analysis revealed that when the number of ACEs increased to four or more, the odds of obesity more than doubled. Among ACEs, emotional abuse had the greatest impact on adult obesity, followed by physical neglect and sexual abuse. As reported in previous studies [138,139,140,141,142]. ACEs are associated with psychological and neuroendocrine disorders that affect self-regulation, appetite, and overall psychopathology. Various aspects of personal life and health are likely to be affected by the intensity of traumatic experiences. It should be noted that severe ACEs, such as sexual abuse, can be associated with post-traumatic stress disorder and metabolic disorder [3,143,144]. The pronounced effect of ACEs on obesity, notably when the number of ACEs exceeds four, and the specific impact of emotional abuse, physical neglect, and sexual abuse on adult obesity rates point to the critical role of tailored interventions. These interventions should not only address the immediate psychological and behavioural consequences of ACEs but also consider the broader socioeconomic contexts and sex-specific vulnerabilities that may influence these outcomes [138,139]. Addressing the cumulative effect of ACEs on health outcomes requires a multi-faceted approach that considers the dose–response relationship and broader societal implications of these experiences. This approach must integrate targeted support and interventions that account for the multifarious nature of ACEs’ impact, emphasising the necessity of breaking the cycle of adversity to mitigate long-term health effects [16,64].

By synthesising these findings, our discussion underscores the need for comprehensive multi-disciplinary strategies that address the intricate web of factors linking ACEs to abnormal BMI. Future research should aim to fill the existing gaps, particularly concerning the less-understood effects of ACEs on underweight status and the specific mechanisms through which socioeconomic and sex factors modulate these relationships.

This study has several strengths. This systematic review and meta-analysis examined the effects of ACEs on a broad spectrum of BMI categories, enriching the field beyond the traditional focus on obesity and overweight. The extensive sample size broadened the generalisability of the findings, making them applicable across diverse populations. This study meticulously examined the variety and intensity of ACEs, providing valuable insights for targeted interventions. Moreover, it delved into psychological and behavioural mediators, highlighting the complex connections between ACEs and BMI and other aspects of BMI, in addition to obesity and overweight.

This systematic review presents a comprehensive analysis of the relationship between ACEs and BMI, highlighting the complexities and challenges inherent in existing literature. However, these findings must be interpreted within the context of several limitations that warrant further consideration. This meta-analysis examined the relationship between ACEs and underweight. Although this relationship can be important, its results have not been extensively reported in previous studies; therefore, only a few studies were included. As cross-sectional studies were also included in this meta-analysis, a causal relationship could not be established.

One significant limitation pertains to the methodological variability across the included studies. Most studies were cross-sectional, limiting the ability to establish causal relationships. Additionally, the reliance on a single method for assessing ACEs in many studies, along with the attrition experienced in longitudinal research, introduces potential biases and reduces the robustness of the findings. Moreover, the heterogeneity in the study designs, participant demographics, and measurement tools for both ACEs and BMI complicates the synthesis of results and necessitates cautious interpretation, which is in line with previous studies [46]. Operationalizing ACEs and BMI is challenging due to diverse definitions and measurement approaches, complicating accurate documentation of ACEs’ effects on obesity. The reliance on self-reported data for ACEs and BMI also introduces recall bias and measurement errors, potentially affecting reliability.

Despite these limitations, this systematic review has several strengths that enhance its contribution to the literature. The rigorous adherence to established protocols, the inclusion of studies spanning several decades, and the absence of restrictions on publication years demonstrate the breadth and depth of the analysis. Moreover, the decision to incorporate studies not included in previous meta-analyses, coupled with the use of multiple databases, underscores the comprehensiveness of the search strategy.

## 5. Clinical Implications

The findings of our systematic review underscore the importance of recognising the significant association between ACEs and adult obesity, as identified in this meta-analysis. This recognition has crucial implications for clinical practice, urging a shift towards a comprehensive bio-psychosocial approach for the management and treatment of obesity. Our findings highlight the need for clinicians to adopt a multi-disciplinary care model that addresses both the physical and psychological effects of ACEs on obesity. This approach entails integrating counselling, nutrition, and management strategies tailored to address the underlying trauma and its contribution to obesity. By acknowledging the complex interplay between ACEs and obesity, clinicians can tailor interventions to meet the unique needs of each patient. Moreover, our review emphasises the importance of trauma-informed practices in clinical settings [145]. Clinicians must be equipped with the knowledge and skills to recognise and address the mental health effects of ACEs and provide a supportive and empathetic environment for patients to discuss their experiences [146]. Implementing trauma-informed care approaches can help build trust and rapport between clinicians and patients, thereby facilitating more effective treatment outcomes.

Additionally, our findings underscore the need to integrate behavioural and lifestyle modifications into obesity interventions. Although traditional approaches to obesity management often focus solely on nutrition and physical activity, our review suggests that addressing the underlying trauma caused by ACEs is also essential for long-term success. Clinicians should consider incorporating trauma-informed strategies into existing obesity interventions, such as mindfulness-based techniques and cognitive-behavioural therapy, to address maladaptive coping mechanisms and emotional eating behaviours [147]. Moreover, the study also highlights the importance of parent–child attachment bonds playing an essential role in causing disordered eating. Therefore, by engaging parents and educating them on improving the parent–child bond, not only can they provide their child with the emotional support required to overcome the negative effects of traumatic experiences but also help protect their children from disordered eating behaviours.

In summary, this systematic review highlights the importance of adopting a holistic approach to obesity management that acknowledges the significant impact of ACEs on physical and mental health. By integrating trauma-informed practices, multi-disciplinary care, and behavioural interventions into clinical practice, clinicians can better support patients in overcoming the complex interplay between ACEs and obesity, ultimately promoting holistic well-being and improving health outcomes.

## 6. Conclusions

The results of this systematic review and meta-analysis show that ACEs are strongly associated with overweight, obesity, severe obesity, and morbid obesity. A relationship exists between ACEs and body weight, with ACEs resulting in increased BMI. Lifestyle choices and stress-induced weight gain are two factors that contribute to ACEs and BMI. Although ACEs and underweight were not significantly correlated, previous studies have limited reporting of this association. This study highlights the need for comprehensive interventions addressing both ACEs and obesity to achieve more effective outcomes. Further research is required to better understand the relationship between ACEs and body weight. Studies should explore the association between ACEs and underweight in greater depth, conduct longitudinal research to establish causality and investigate the underlying mechanisms. By understanding these aspects, targeted interventions and strategies can be developed to prevent and manage obesity in individuals affected by ACEs. Therefore, healthcare professionals and policymakers should prioritise ACEs in their strategies to prevent and manage obesity and promote healthier outcomes for individuals affected by ACEs.

## Data Availability

The datasets generated and/or analysed during the current systematic review and meta-analysis are derived from publicly available resources. The statistical data supporting the findings of this study are available for the corresponding author upon reasonable request.

## Supplementary Materials

The following supporting information can be downloaded at: https://www.mdpi.com/article/10.3390/children11081015/s1, Supplementary Figure S1: Flowchart diagram. Supplementary Figure S2. Meta-analysis of adverse childhood experiences and morbidly obese, severe obesity, and underweight. Supplementary Figure S3. Meta-analysis of ACEs 1 and Underweight, Overweight, Obesity, severe obesity, and morbidly obese. Supplementary Figure S4. Meta-analysis of ACEs 2 and Underweight, Overweight, Obesity, and severe obesity. Supplementary Figure S5. Meta-analysis of ACEs 3 and Underweight, Overweight, Obesity, and severe obesity. Supplementary Figure S6. Meta-analysis of ACEs ≥4 and Underweight, Overweight, Obesity, severe obesity, and morbidly obese. Supplementary Figure S7. Meta-analysis of emotional abuse and emotional neglect and Body Mass Index class. Supplementary Figure S8. Meta-analysis of physical abuse and Body Mass Index class. Supplementary Figure S9. Meta-analysis of physical neglect and Body Mass Index class. Supplementary Figure S10. Meta-analysis of sexual abuse and Body Mass Index class. Supplementary Figure S11: Funnel plot of adverse childhood experiences and obesity. Supplementary Figure S12: Funnel plot of adverse childhood experiences and overweight. Supplementary Figure S13: Funnel plot of adverse childhood experiences and overweight/obesity. Supplementary Table S1: Studies included in the meta-analysis.

## Appendix A. Keywords Used for Systematic Search in PubMed, Web of Science, and Scopus Search until August 2023

**Table:**

Search	Query
PubMed	1173
#1	Adverse Childhood Experiences [Mesh] OR Adverse Childhood Experiences [Text Word] OR Childhood Trauma [Text Word] OR Traumatic Childhood Experiences [Text Word] OR Early Life Stress [Text Word] OR Childhood abuse [Text Word] OR Child Abuse [Mesh] OR Child Abuse [Text Word] OR Child Mistreatment [Text Word] OR Child Neglect [Text Word] OR Sexual Child Abuse [Text Word] OR Emotional abuse [Mesh] OR Emotional abuse [Text Word] OR Physical abuse [Mesh] OR Physical abuse [Text Word]
#2	obesity [Mesh] OR obesity [Text Word] OR body weight [Mesh] OR body weight [Text Word] OR overweight [Mesh] OR overweight [Text Word] OR underweight [Mesh] OR underweight [Text Word] OR waist circumference [Mesh] OR waist circumference [Text Word] OR waist-hip ratio [Mesh] OR waist-hip ratio [Text Word] OR body mass index [Mesh] OR body mass index [Text Word] OR Body Size [Mesh] OR Body Size [Text Word]
Final	#1 AND #2
Scopus	1928
#1	“Adverse Childhood Experiences” OR “Childhood Trauma” OR “Traumatic Childhood Experiences” OR “Early Life Stress” OR “Childhood abuse” OR “Child Abuse” OR “Child Mistreatment” OR “Child Neglect” OR “Sexual Child Abuse” OR “Emotional abuse” OR “Physical abuse”
#2	“obesity” OR “body weight” OR “overweight” OR “underweight” OR “waist circumference” OR “waist-hip ratio” OR “body mass index” OR “Body Size”
Final	#1 AND #2
Web of Science	4654
#1	TS = (Adverse Childhood Experiences OR Childhood Trauma OR Traumatic Childhood Experiences OR Early Life Stress OR Childhood abuse OR Child Abuse OR Child Mistreatment OR Child Neglect OR Sexual Child Abuse OR Emotional abuse OR Physical abuse)
#2	TS = (Obesity OR body weight OR overweight OR underweight OR waist circumference OR waist-hip ratio OR body mass index OR Body Size)
Final	#1 AND #2

## Appendix B. Quality Assessment

**Table:**

Type of Bias	Criteria Definition	Classification (Potential for Bias)
Selection bias	Sampling method of the study population, representativeness (response rate, difference between responders and non-responders, investigate and control of variables in case of difference between responders and non-responders)	**Low**: Target population defined as representative of the general population or subgroup of the general population (specific age group, women, men, specific geographic area, and specific occupational group) and response rate is 80% or more. **Moderate**: Target population defined as somewhat representative of the general population, a restricted subgroup of the general population, response rate 60–79%. **High**: Target population defined as “self-referred”/volunteers, response rate less than 60%.
Confounders	Matching two groups Stratification Statistical analysis	**Low**: Controlled for most potential confounding factors including age and sex. **Moderate**: Controlled for few potential confounding factors, including both age and sex. Adjusted but variables are unknown. **High**: Not controlled for both age and sex, or controlled for less than two confounding factors.
Performance bias	Valid and reliable assessment of exposure	**Low**: Valid and reliable tools for data collection. **Moderate**: Valid and not reliable tools or reliability is not described. A series of questions. **High**: Without validity and reliability or both reliability and validity are not described. Unspecified measure. Only one or two items used.
Data collection method	Valid and reliable assessment of outcome	**Low**: Body mass index was Measured by Digital height and weight measurement. **Moderate**: Body mass index was self-reported or other reported or unknown.
Withdrawals and drop-outs	Withdrawals and drop-out rates Size of missing data	**Low**: Follow up participation rate of 80% or higher or missing data on less than 20%. **Moderate**: Follow up participation rate of 60–79%, or missing data on 20–40%. **High**: Follow up participation rate of less than 60%, or missing data on more than 40%.
