# Supplementary material for: Adverse Childhood Experiences and Risk of Abnormal Body Mass Index: A Global Systematic Review and Meta-Analysis
Source: Children (Basel). 2024 Aug 20;11(8):1015. doi: 10.3390/children11081015 (PMC11352292; doi:10.3390/children11081015)
Supplement: Supplementary file 1 [file children-11-01015-s001.zip › children-3116480-supplementary.pdf]

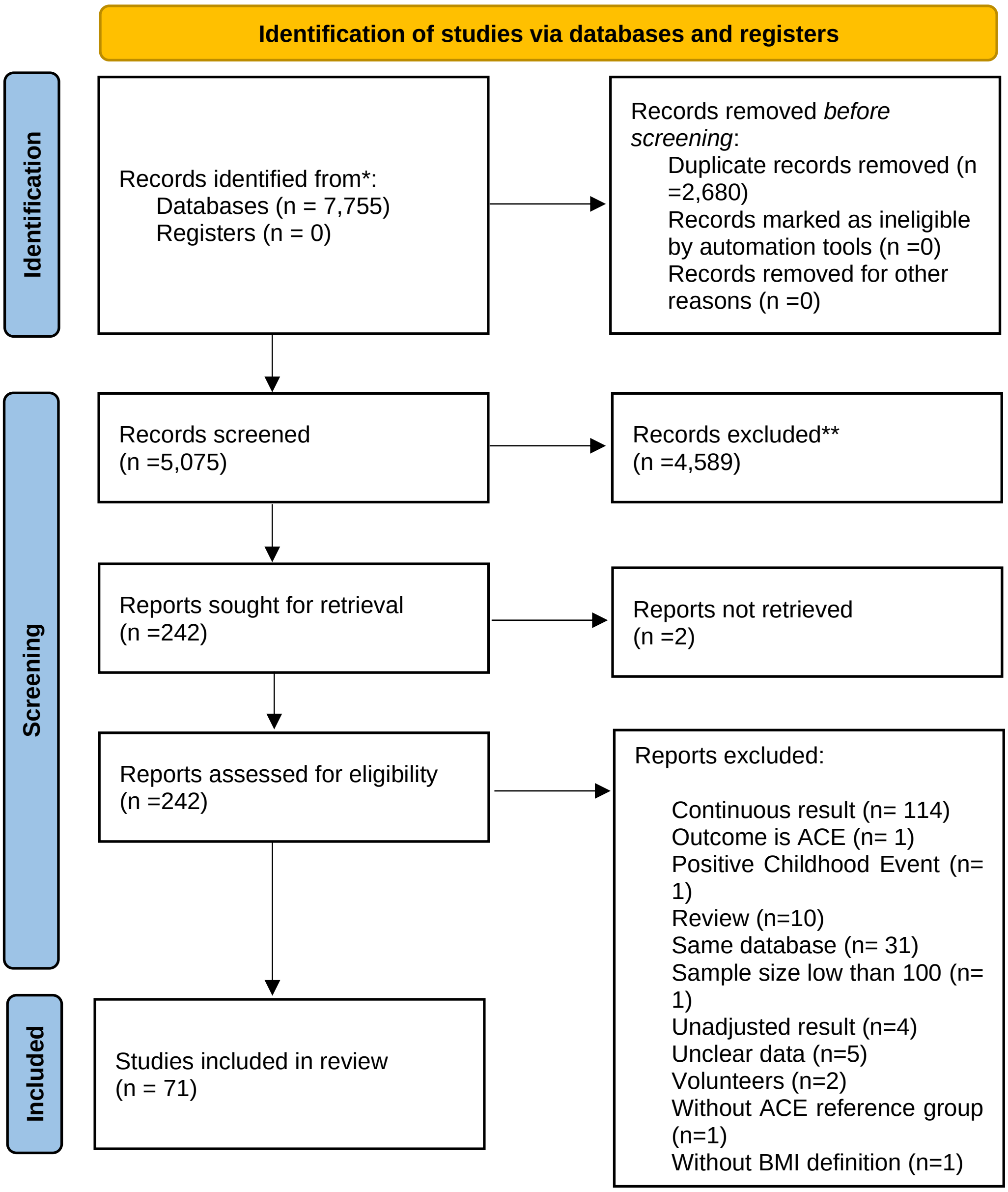

Supplementary Figure 1: PRISMA Flowchart diagram

From: Page MJ, McKenzie JE, Bossuyt PM, Boutron I, Hoffmann TC, Mulrow CD, et al. The PRISMA 2020 statement: an updated guideline for reporting systematic reviews. BMJ 2021;372:n71. doi: 10.1136/bmj.n71

For more information, visit: http://www.prisma-statement.org/

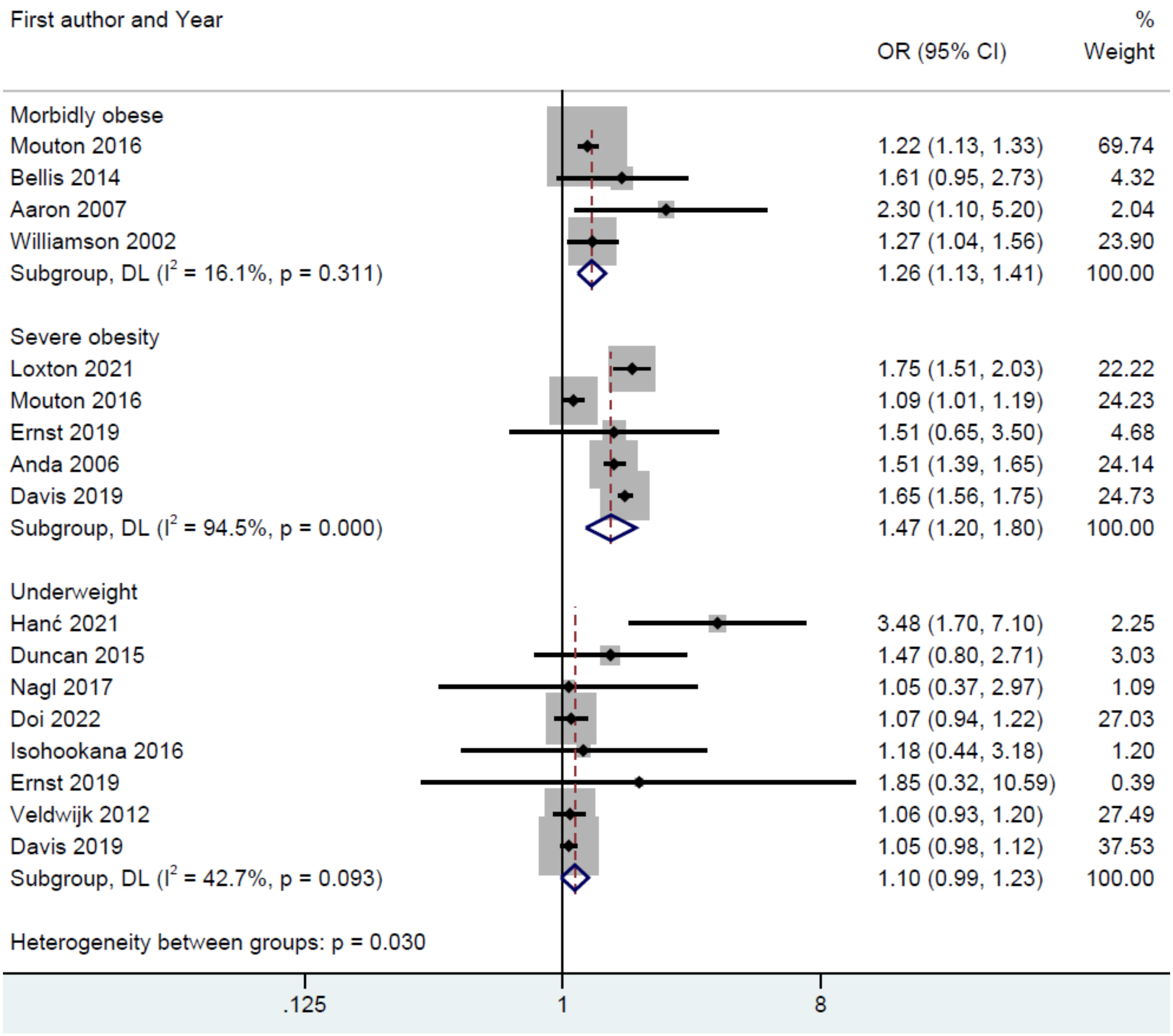

Supplementary Figure2. Meta-analysis of adverse childhood experiences and morbidly obese, severe obesity, and underweight

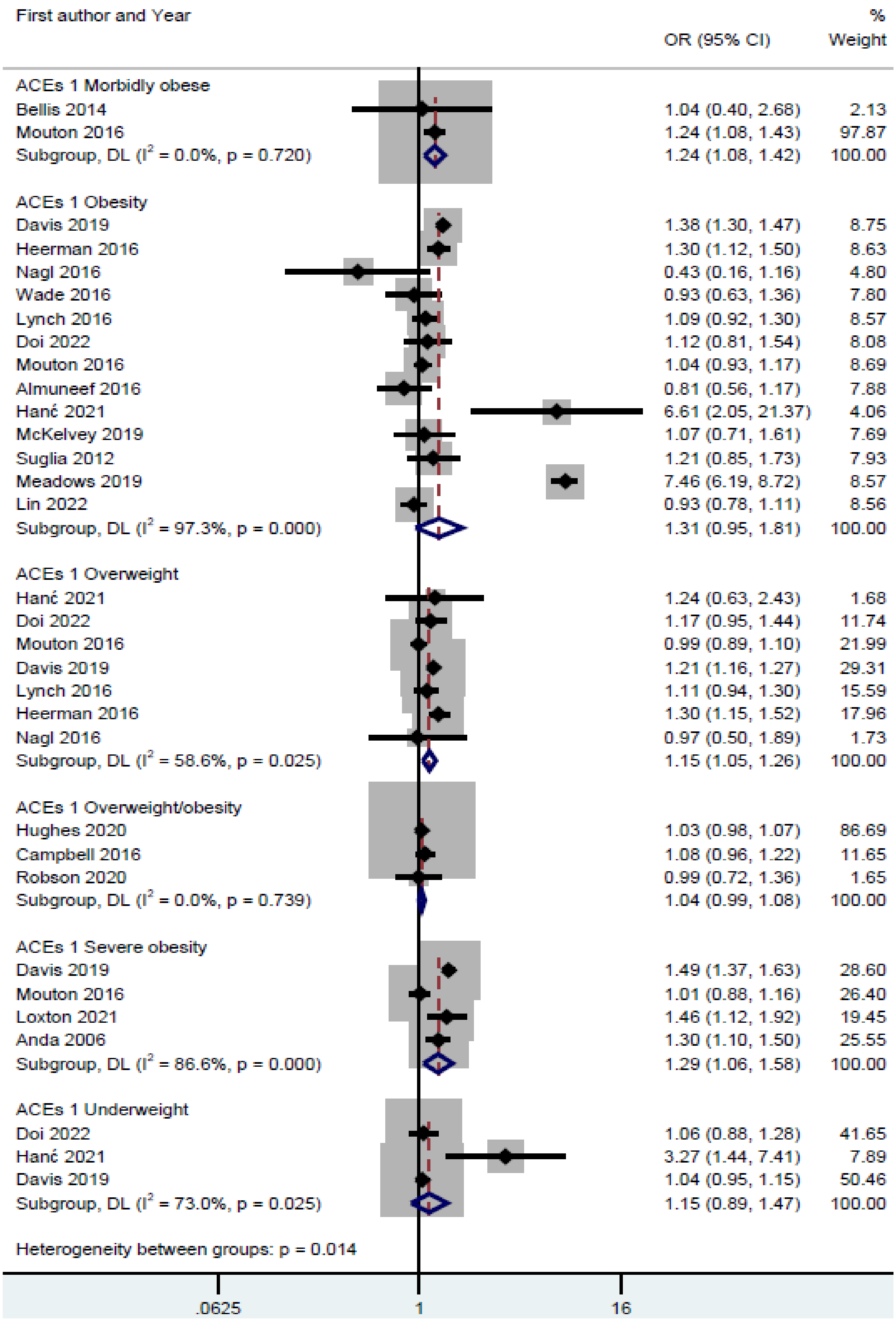

Supplementary Figure3. Meta-analysis of ACEs 1 and Underweight, Overweight, Obesity, severe obesity, and morbidly obese

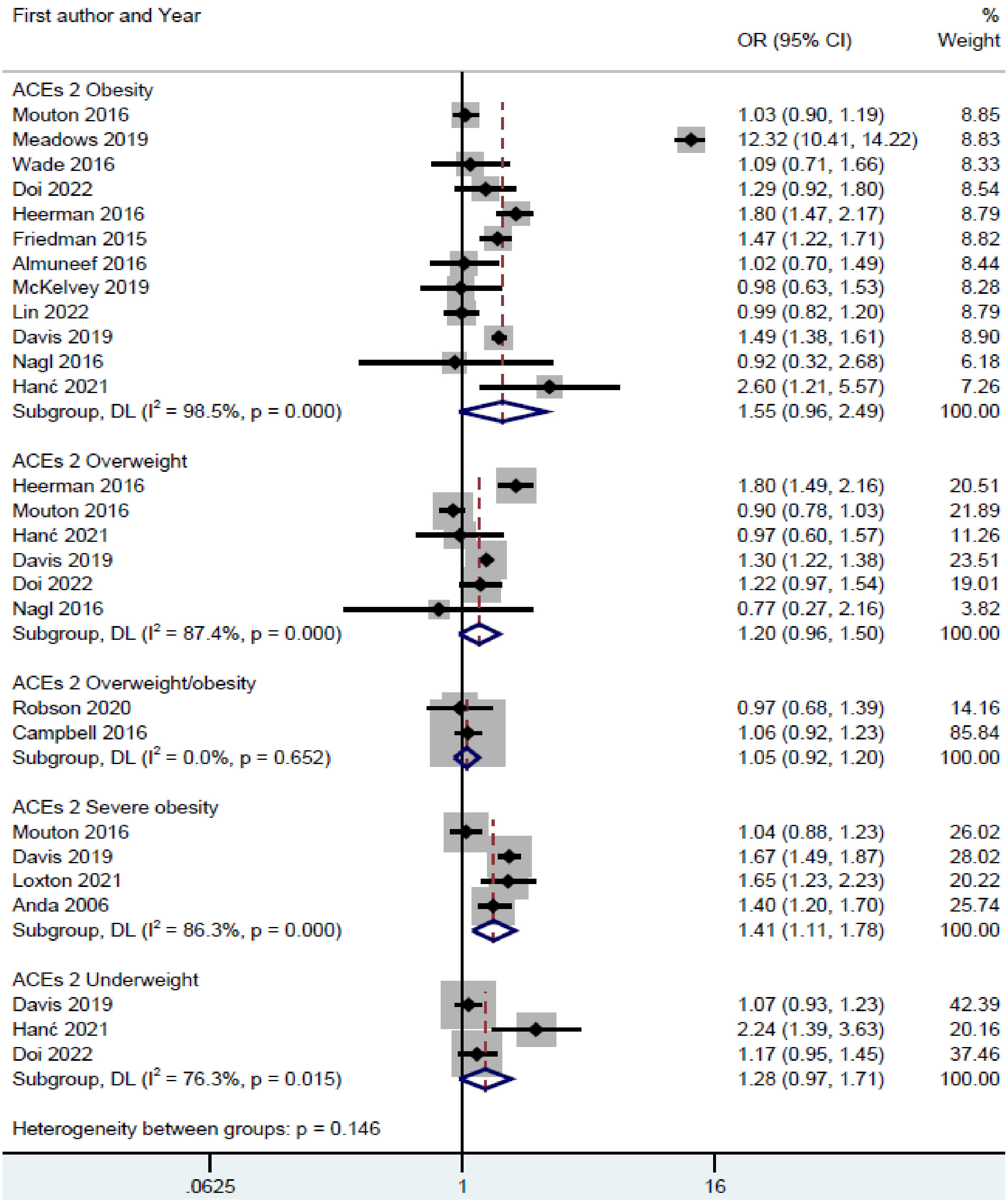

Supplementary Figure4. Meta-analysis of ACEs 2 and Underweight, Overweight, Obesity, and severe obesity

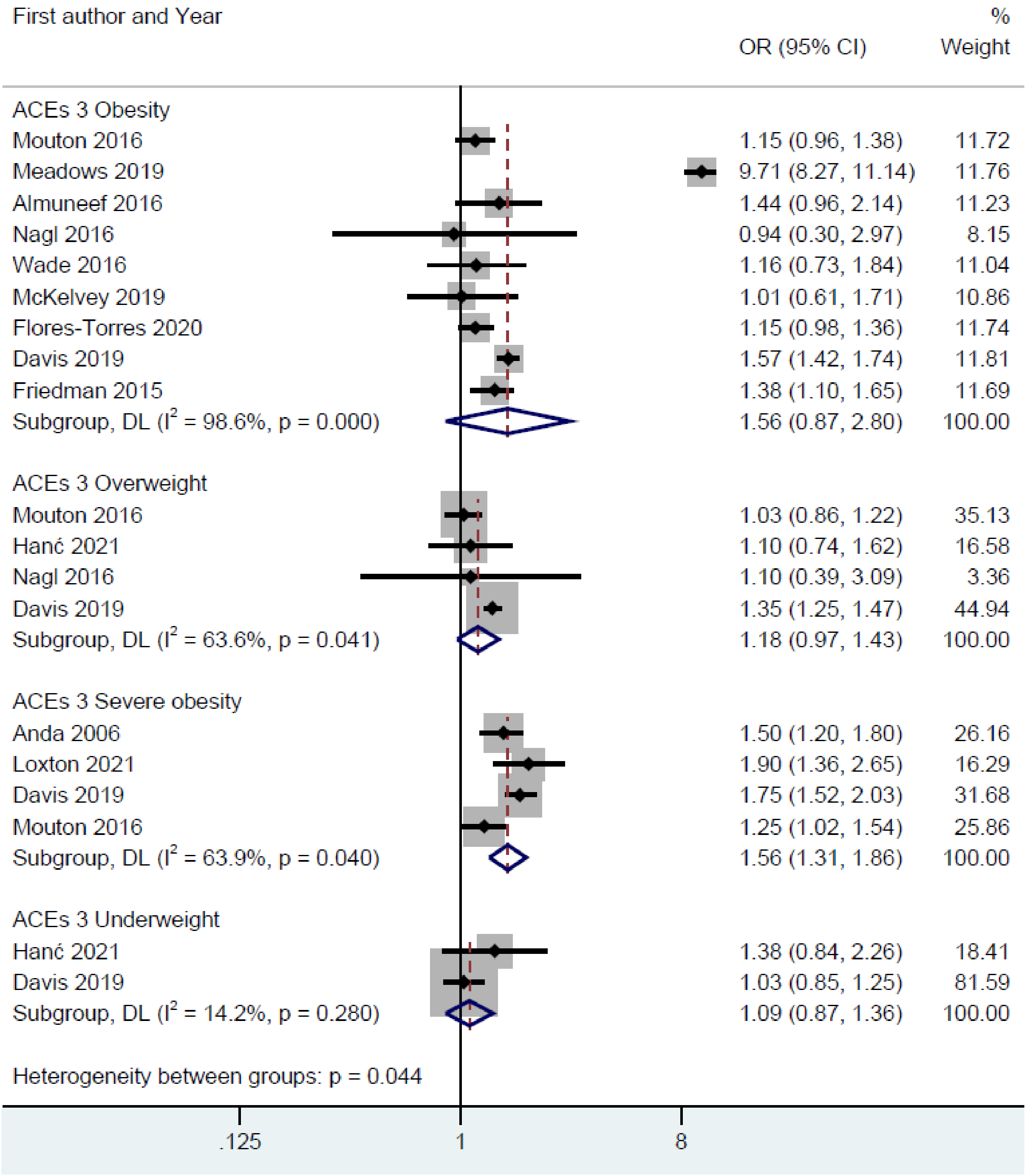

Supplementary Figure5. Meta-analysis of ACEs 3 and Underweight, Overweight, Obesity, and severe obesity

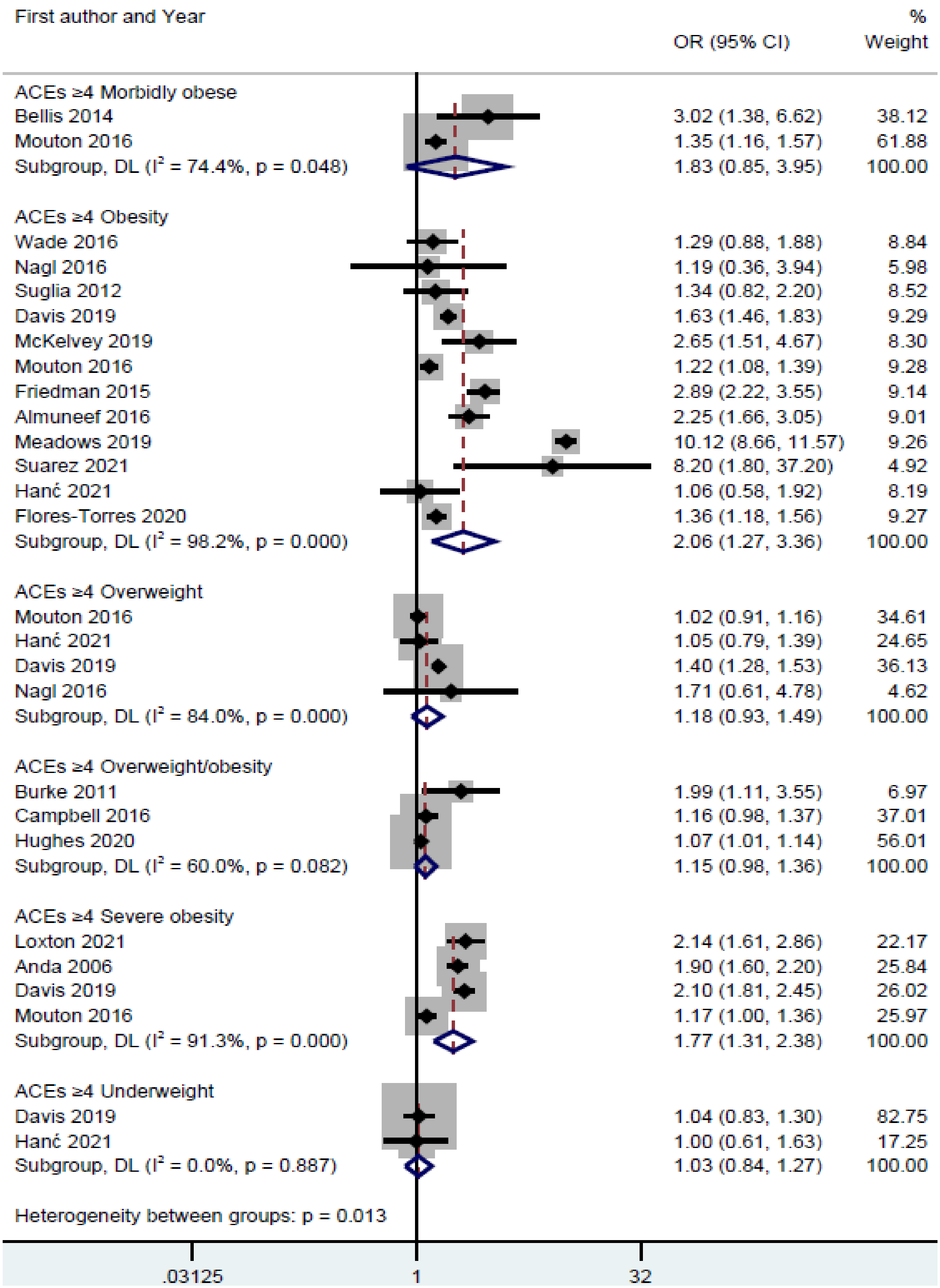

Supplementary Figure6. Meta-analysis of ACEs ≥4 and Underweight, Overweight, Obesity, severe obesity, and morbidly obese

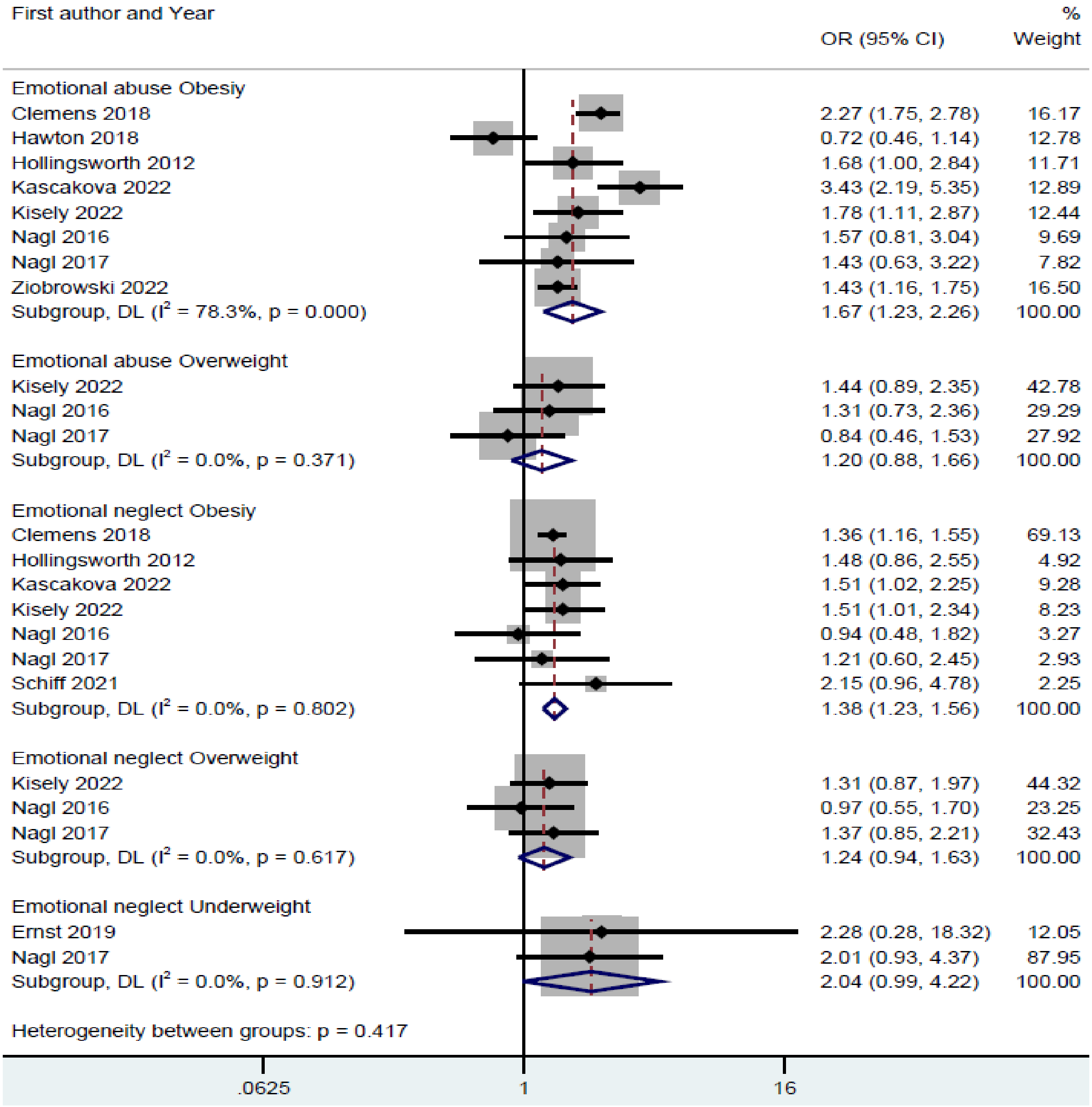

Supplementary Figure7. Meta-analysis of emotional abuse and emotional neglect and Body Mass Index class

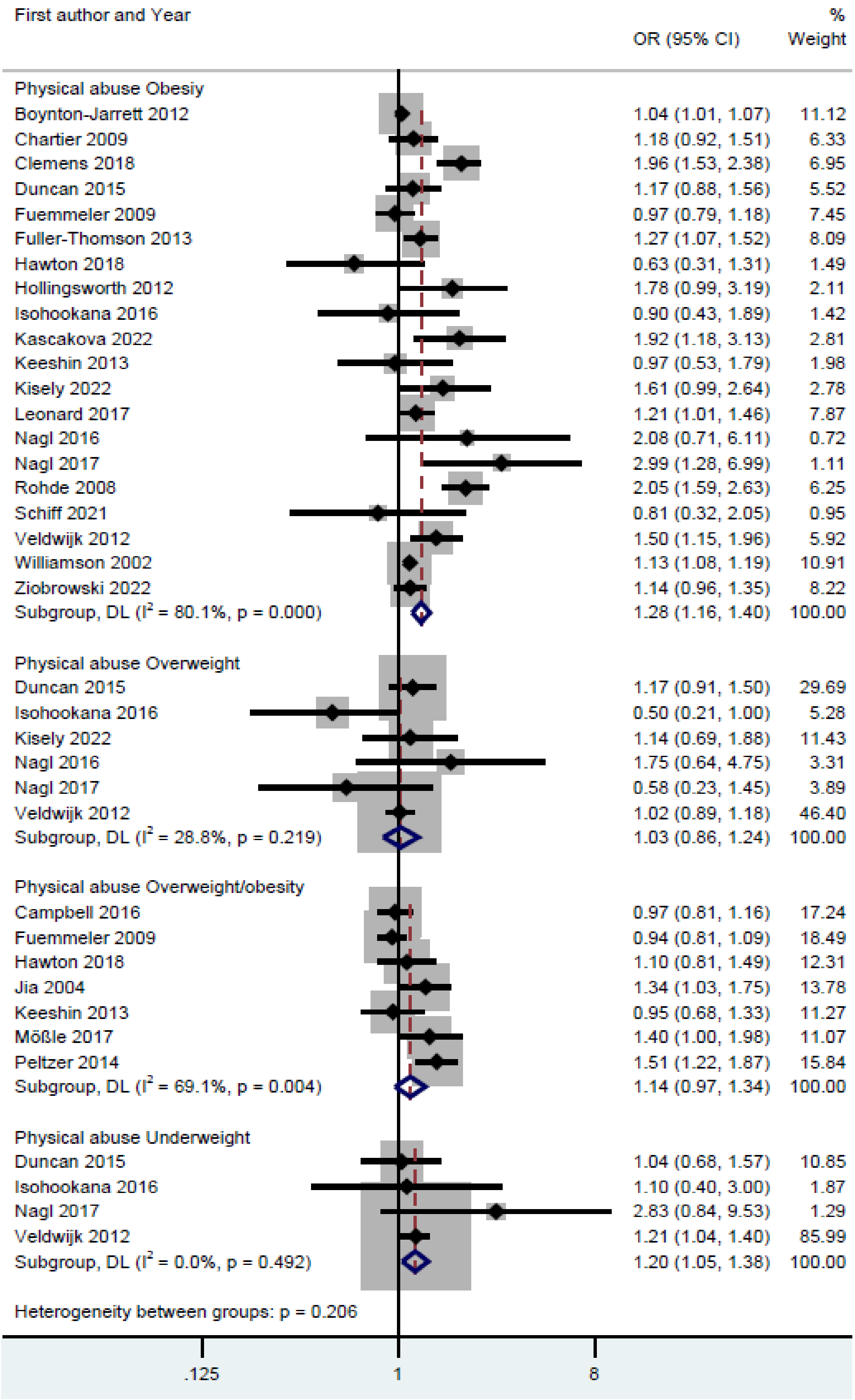

Supplementary Figure8. Meta-analysis of physical abuse and Body Mass Index class

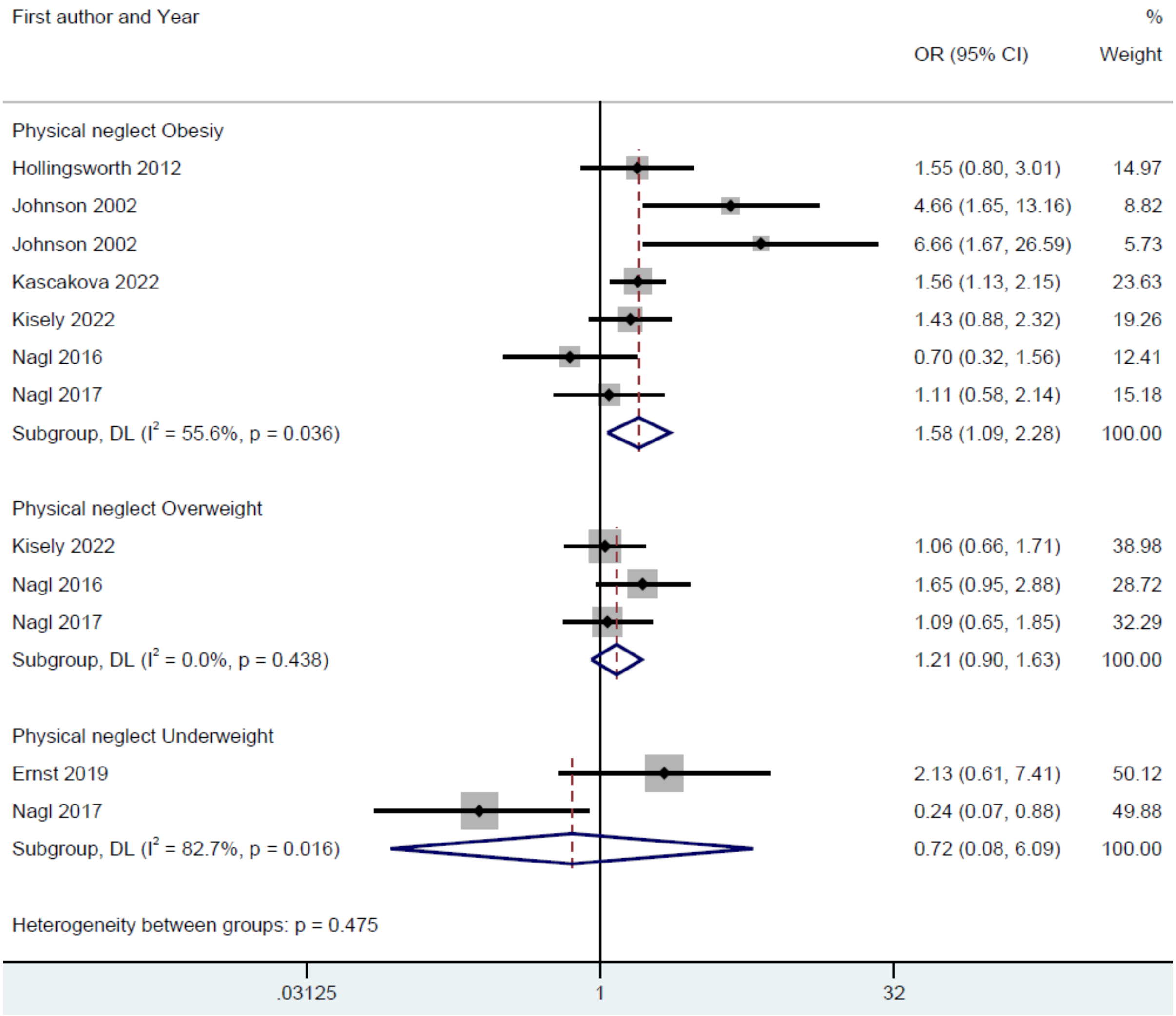

Supplementary Figure9. Meta-analysis of physical neglect and Body Mass Index class

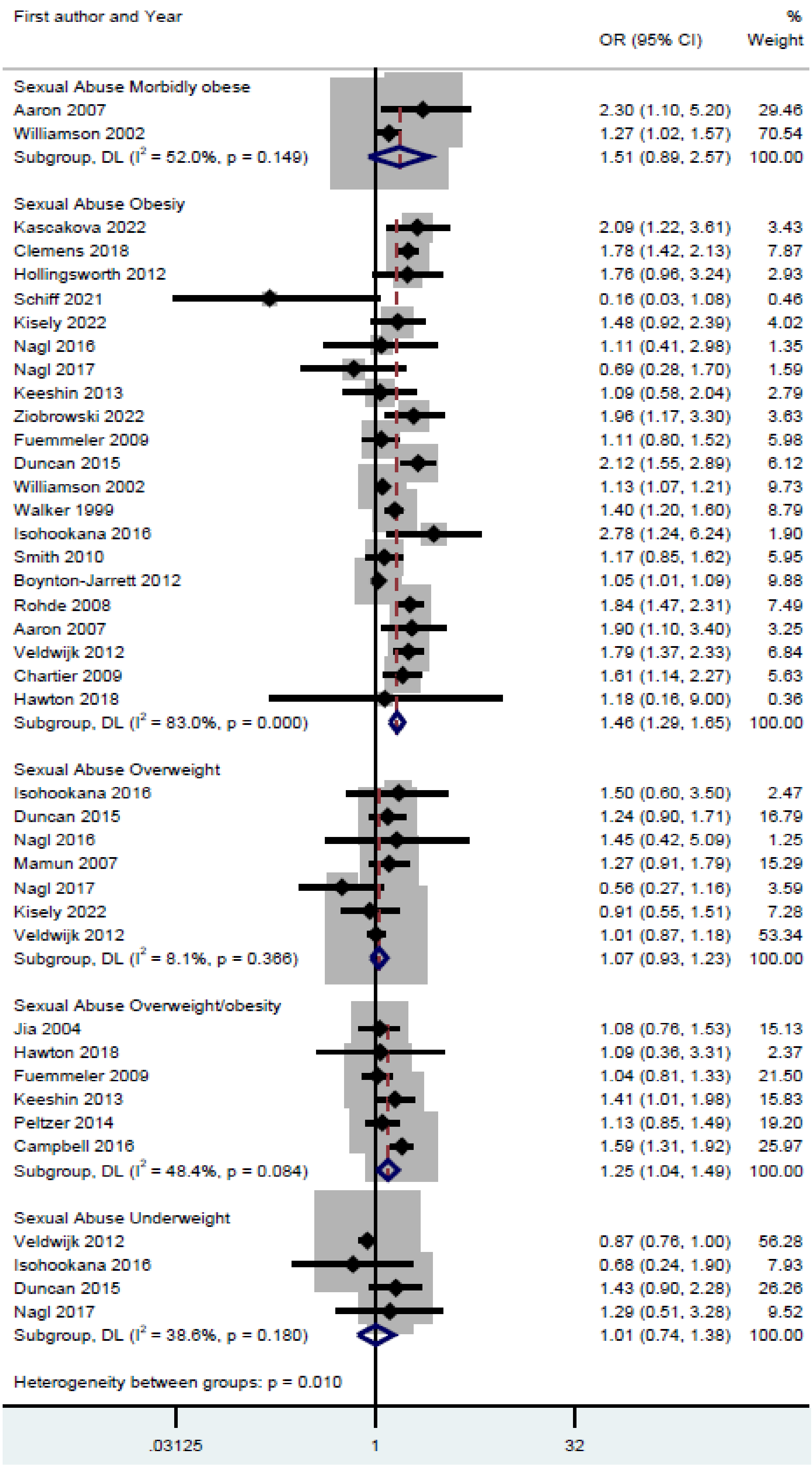

Supplementary Figure10. Meta-analysis of sexual abuse and Body Mass Index class

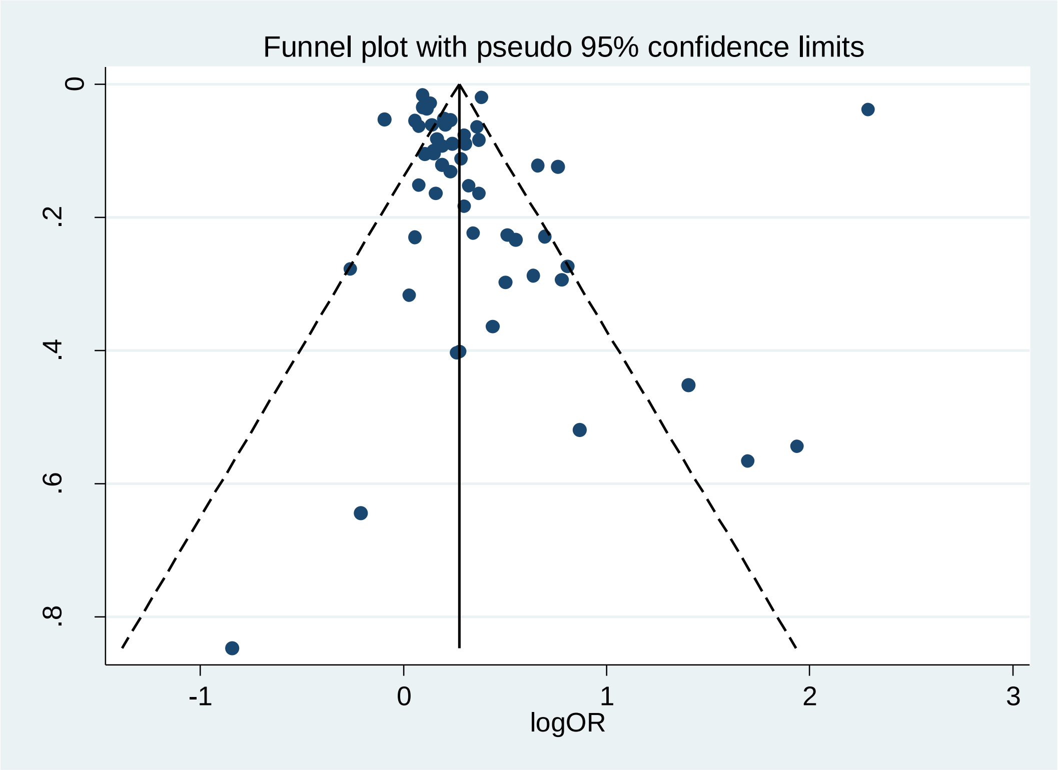

Supplementary Figure 11: Funnel plot of adverse childhood experiences and obesity

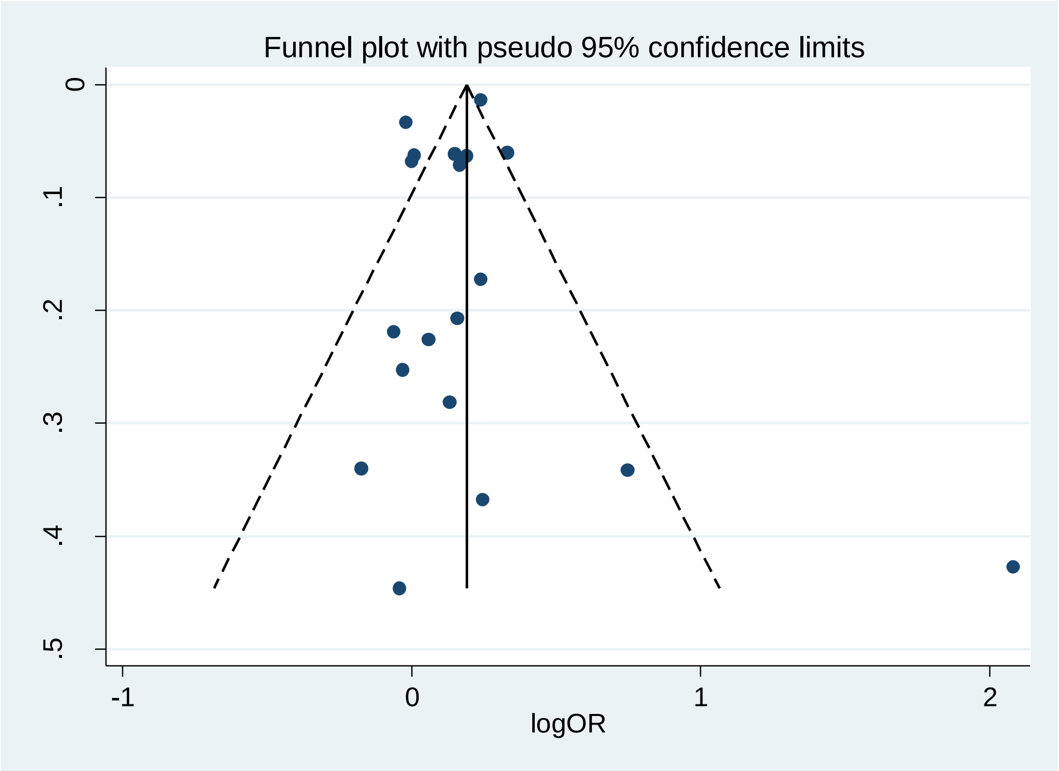

Supplementary Figure 12: Funnel plot of adverse childhood experiences and overweight

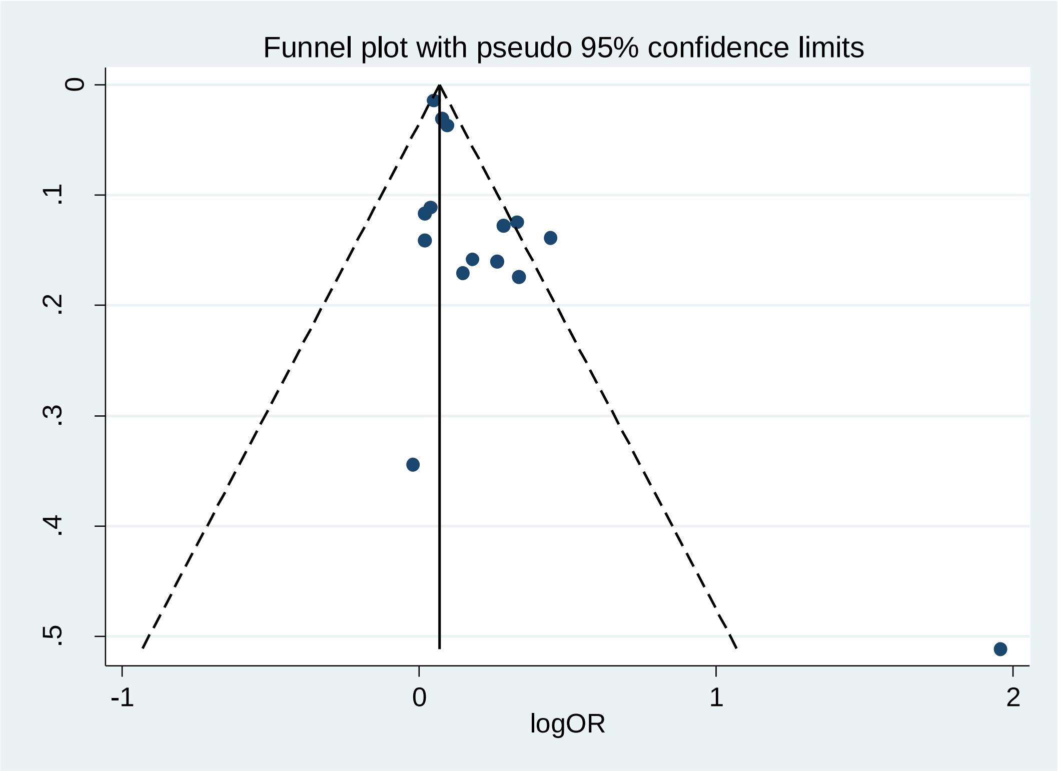

Supplementary Figure 13: Funnel plot of adverse childhood experiences and overweight/obesity
